# Bridging the knowledge gap between technology and business: An innovation strategy perspective

**DOI:** 10.1371/journal.pone.0266843

**Published:** 2022-04-14

**Authors:** Pejman Peykani, Mehdi Namazi, Emran Mohammadi

**Affiliations:** 1 School of Industrial Engineering, Iran University of Science and Technology, Tehran, Iran; 2 School of Progress Engineering, Iran University of Science and Technology, Tehran, Iran; Sichuan Agricultural University, CHINA

## Abstract

Decision-makers (DMs) are not sufficiently exposed to concepts such as efficiency and risk in innovative activities from the perspective of organizational strategy. The challenges become even greater when these DMs lack expertise in technology and deal with uncertain circumstances. In this sense, exchanging expert knowledge between DMs and technical teams will strengthen the link between technology planning and strategic management. The purpose of this study is to bridge the knowledge gap between these two groups. It introduces a framework to translate the organization’s strategy into technological decisions at an acceptable innovation risk level. This framework considers aspects such as knowledge, type of innovation, and innovation process. This study focuses on determining whether activities should be accepted or rejected by examining the uncertainty and efficiency of innovation. It also introduces a novel perspective on the hybrid "success-failure" uncertainty of innovation, and a new measure called "efficiency probability," which DMs and technology developers can use to intuitively engage in the innovation process. This paper seeks to propose a practical strategy map for new product development under uncertain conditions. To achieve this goal, the Fuzzy Front-End (FFE) concept, fuzzy data envelopment analysis (FDEA) model, and adjustable possibilistic programming (APP) approach are applied. The results of this study indicate that innovative activities typically have low efficiency and high uncertainty. Therefore, the decision to implement or abandon them requires reviewing and balancing the goals and strategic approach of the organization with technological and business features.

## I. Introduction

The initial phase of the innovation process is the Fuzzy Front-End (FFE). It begins with spotting new opportunities, followed by new business cases and ideas, and closes when companies decide to execute an innovative project in the succeeding stages of the innovation process [1]. Strictly speaking, FFE embraces all activities accomplished ahead of any formal development of a product’s details or launch [2].

The front-end is also strategically significant since it assures the coherence between new products and services to business goals by attaching the innovation process to business strategies [3]. The core of this early phase in the innovation process is decision-making, where agreement is achieved on many important issues concerning features, position, performance, technology, resources, and portfolio alignment of new products and services. Therefore, the abovementioned decisions are packed into proposals and business plans, assisting go/no-go decisions for the succeeding stage of the innovation process, which is development and commercialization [4]. Van Aken & Nagel [5] claimed that the FFE has a high level of creativity, ambiguous initiation, various inputs, vague process, and spontaneous participant involvement.

Hence, the FFE is roughly an iterative and ambiguous process. In contrast, the development and commercialization level are relatively a straight and clear process. The quality of FFE is very important for the next phase of the innovation process as product characteristics, constraints, and advantages are established in this phase [6], so it has a high potential for cost and time savings, quality improvements, and product adjustments when the actual product development and commercialization begins. Table 1 shows the differences between the FFE phase and the development and commercialization phase [7]:

**Table 1:** Fuzzy front-end vs development and commercialization phase.

Feature	Fuzzy Front-End	Development & Commercialization
Type of information	Qualitative, informal, and inaccurate.	Quantitative, formal, and accurate.
Cost of project kill	Small.	Considerable.
Width and depth	Wide but lean.	Narrow but deep.
Output	A proposal.	A product.
Budgeting	Small but varying.	Large and planned.
Nature of the system	Hard to plan, eureka occasions, tentative, often disordered.	Organized, disciplined with clear goals and plans.
Management approaches	Informal, tentative, creativity-oriented.	Formal, organized.

Various FFE models are presented in the literature. Koen et al. [2] illustrated the uncertain and iterative outlook of the FFE in a circular shape, implying that actions in the model can iterate through five steps. However, their model was limited to just one idea selection step and lacked a decision gate as the quality–control checkpoint [8]. Therefore, Cooper [9] argued the importance of the go-to development gate as the last step, where an idea could be dropped before starting a costly development phase. Hence, the go-to development gate was added to the model of Koen, et al. The go-to development gate involves a review team to determine Go/ Kill/ Hold/ Recycle decisions, approved action plans, a list of deliverables, and some criteria for product development [9].

The decision-making and awareness at the go-to development gate have a dual nature. On one hand, the technical managers are aware of weaknesses, capabilities, and limitations of technology development units as well as possible. On the other hand, the rationale of the organization is at the hands of senior executives who explain the strategy and prefer a lighthouse, to determine the direction of the organization in the turbulent sea of business and technology. For a technology-driven company, strategy determines the nature of innovative activities. Although managers know that technology planning and strategic management are inseparable, a large part of their focus is naturally on management issues. Conversely, technical people innately focus on technology.

Hence providing tools and processes to facilitate communication between these two groups can potentially enhance the connection between technology planning and strategic management. The review team exploits the company’s approval level to make a Go/ Kill/ Hold/ Recycle decision for every project, considering various internal and external conditions that may influence on company’s approval level. Kim & Wilemon [10] provided examples of these circumstances that are corporate strategy and culture, pressure for development, technology readiness, available resources, project risk, risk-taking tolerance, and the ambiguity level at the end of the FFE. Due to the compilation of the above situations, it can be concluded that the review team aims to maximize the value of innovative ideas and projects, which may generate acceptable inherent risk.

An abstraction to this decision-making orientation is maximizing innovation efficiency under uncertainty. As explained before, FFE has excessive ambiguity that leads to qualitative, informal, and rough information for decision-makers. While there are many studies on the selection of innovative projects, new product development portfolios, and R&D projects [11], they are mainly focused on rational decision-making and are somehow deficient in empirical data on the function and performance of political and intuitive decision-making. The findings reveal that we face an intuitive type of decision-making at the early phase of the innovation process followed by political approaches while the later phase of the innovation process demands rational decision-making. That is why Eling et al. [12] marked intuition as an important factor influencing the process of decision-making at the go-to development gate.

The review team is responsible for making predictions and commenting on decisions, but their intuitions may be problematic where the decision-making process is based on the power or the majority votes. Subsequently, precious intuitions could be vanished or may have to struggle with the power of the majority [12]. As a result, political and rational decision-making could hinder intuition. Also, according to Hart et al. [13], the most important criteria for the review team are technical feasibility, product performance, and customer acceptance. Essentially, the review team is facilitated by a non-voting member and involves senior people who allocate the resources for the project team.

It is suggested that the review team shall not only involve people with creativity and optimistic personas [2,7], but also critical members [14]. These findings convey the importance of intuitive decision-making and its vulnerability under the shadow of political and rational approaches. This is in line with the study of Riel et al. [15] where they focused on the importance of valuation indices for ideas in pursuance of generating innovation that supports idea selection and prioritization in basic decisions.

On one hand, the review team contains the appropriate combination of seniority, but it does not necessarily involve the project team members [14]. Hence, there is a gap between the development team and the DMs. The fact is that the decisions about innovative activity made by the review team play an entrepreneurial role within the firm. Therefore, just like any other entrepreneur, they need to know about the risks and rewards of that activity. The communication between the development and review team will lead to an understandable and credible position of each project that considers the innovative aspect of corporate strategy. On the other hand, the review team has to signal the innovative orientation of corporate strategy down to the organization, especially to the development team to broadcast coherency between corporate strategy and innovative activities within the firm.

The decision-makers in the review team are faced with the problem of connecting between the technical units and the decision-making body so that the organization’s innovation strategy is well implemented. They are seeking a mechanism that can create a convergence between these two groups so that the inherent uncertainty of innovation and the innovation activities of the organization could be aligned with the innovation strategy. Besides, clarifying the hidden awareness of uncertainty in the technical body of the organization is of great importance.

To address the above decision-making challenges at the go-to development gate, this study has the following research questions:

- How can we harvest the intuitions of both the development and the review team at the go-to development gate?
- How can we include both optimistic and pessimistic intuitions in the decision-making process?
- How can we align the above decisions with corporate strategy?

To answer the above questions, this research focuses on measuring the efficiency of innovation under uncertainty so that the innovative activities can be aligned with the organization’s innovation strategy.

Researchers have already combined data envelopment analysis techniques with other concepts to provide pragmatic analyzes of the innovation-related topic [16,17]. Combining fuzzy theory with data envelopment analysis is proved to be an advantageous tool to deal with uncertainty [18,19]. The Fuzzy Front-End of product development always confronts uncertainty, so we propose an efficient model based on an adjustable fuzzy data envelopment analysis (AFDEA) for intuitive involvement and decision making within a fuzzy environment. Note that in the AFDEA model, DMs can easily set different optimistic-pessimistic attitudes merely by adjusting a parameter. As a final step, a case study from the Iranian industry will illustrate the applicability of the proposed approach.

The structure of this study is as follows: An introduction to the problem is presented in section one. Section 2 explores the research background on the strategic evaluation of innovative activities under uncertainty. Section 3 provides an introduction to fuzzy measures and fuzzy data envelopment analysis. The research methodology is presented in Section 4 followed by Section 5 which presents empirical results. Summary and conclusions are presented in Section 6.

## II. Research background

In 1989, Daniela presented a comprehensive review of the different ways to strategically evaluate innovative projects and found that in a highly competitive world, organizations use methods with significant strategic benefits [20]. In the same year, Sanchez explored the role of project evaluation techniques in defining a company’s technology strategy. He classified the strategies into four groups: planning, economic, market, and technical strategies. Sanchez asserts that using an economic strategy requires pre-defined selection criteria and applying selection methods is not flexible and in the case of market strategy, evaluation methods only help prioritize projects. He also concluded that decisions made by companies that use a technical strategy are solely based on the technical competence of the various projects that are intended to be implemented [21].

The first empirical research on the relationship between innovation and strategy in various articles indicates three main objectives for successful companies [22] in innovation: maximizing portfolio value, achieving a balance between innovation practices, and alignment with strategy [23,24]. This, as later developed for the fourth purpose: selecting the right number of innovative activities [25]. The results highlight the importance of coordinating strategic decisions and innovation. However, it was somewhat unknown to understand how this link may be realized. Various strategies for managing innovative activities are possible [26] and companies that openly express areas of strategic focus in their innovation portfolios [27] or the process of managing the portfolio of ideas by managing the innovation portfolio [28] have more fruitful results. To align the innovation portfolio with relevant strategic objectives, specific tools and methods such as strategic buckets have been proposed [29]. However, experimental results show that companies that have moved from purely financial instruments to strategic tools are more successful in innovation [22].

The above studies focused on different aspects of managing the innovation portfolio, but they lack the most required insight into the actual implementation of strategic goals in the decision-making process. This is because ambiguity is at the core of innovation and often leads to risk and uncertainty. Most researches in this area include a combination of models, methods, and techniques that support the evaluation of innovative activities, but few show the relationship between innovation and the company’s strategy of uncertainty. Cheung et al. [30] pioneered using confusing choices to fill this gap in technology-based companies, but they focused only on the ambiguity of knowledge. Wang et al. [31] proposed a framework for aligning risk management with technological innovation with organizational strategy, but they ignored the state of innovation which is at the heart of the company’s strategy.

Many studies on the evaluation of technological innovation based on the company’s strategy used this framework. For example, Herfert & Arbige [32] introduced an iterative process and showed how innovation is in line with corporate strategy. However, to evaluate innovations regarding corporate strategy they focused only on business and portfolio management tools. Once again, Rhéaume & Gardoni [33] conducted a comprehensive study on the relationship between a company’s strategy and management of innovative activities like developing new products. Although, they failed to provide any guidelines or frameworks for translating innovation strategies into resource allocation.

For incremental ideas, Ansoff et. al [34] tried to fill this gap and used "strategic situation" to manage technological innovation. They defined the "Incremental" and "Continuous" Strategic modes. Although their classification seems inherent, it does not provide a clear concept of whether or not to issue a ruling on innovation. That is why they used this classification to make decisions about innovative management styles rather than their evaluation. The go-to development gate is a bridge between the blurry world of fuzzy front end and clarifies the world of product development. The multifunctional nature of the project team results in a multifunctional review team. This fact results in a better decision-making capability for the review team.

The review team could also involve suppliers, technology partners, investors, and customers [14]. However, the seniority of the team members depends on the type and importance of the idea. For incremental ideas, the review team comprises mid-management while for radical and important ideas the senior management also is involved [9]. Rhéaume & Gardoni [33] focus was on explaining the relationship between company strategies and managing innovative activities. They paid almost no attention to other aspects. Using business management tools, Herfert & Arbige [32] came up with a good approach to select innovative activities but he did not mention how the acceptable level of innovation risk is determined by the organization’s strategy.

Wang et al. [31] provided a framework for managing risk in innovative activities with an organizational strategy that takes into account knowledge considerations and the type of innovation. However, he did not specify the status of innovative activity in the organization’s strategy. In other words, he argued that the risk management framework can balance the risk of activities according to the company’s strategy, but he did not specify which strategic approach each company is taking should take or which activities are likely to be appropriate given the company’s strategic approach.

Cheung et al. [30], like Rhéaume & Gardoni [33], considered just one indicator and ignored other indicators. He analyzed the ambiguities of knowledge and provided a way to strategically select them. Chao & Kavadias [29] only paid attention to the balance between radical and continuous innovation and did not offer a way to translate strategy into an acceptable level of ambiguity in the organization.

Table 2 represents a summary of previous research on strategic evaluation of innovative activities under uncertainty. This table, categorizes studies based on innovation aspects that they have considered. These aspects include knowledge, translating strategy to level of risk tolerance, risk management, portfolio management guideline, innovation process, innovation type, and the uncertainty approach. As seen in this table, only Ansoff and his colleagues could cover all aspects, nevertheless, they did not issue a clear go/no go verdict for a given project. There is a major gap in all previous studies, as they have examined innovative projects against corporate strategy, but none could combine the knowledge and intuition of both the review team and the development team. In other words, they could not bring the management and technical teams together to reach a better decision.

**Table 2:** Major pieces of research on strategic evaluation of innovative activities under uncertainty.

	Researchers	Considering Knowledge	Translating Strategy to Risk Tolerance	Possible Risk Management	Portfolio Management Guideline	Considering the Innovation Process	Considering the Innovation Type	Uncertainty Approach
Critique	Chao & Kavadias [29]		✓	✓	✓		✓	Indirect
	Only focused on the balance between radical and continuous innovation							
	Cheung et. al. [30]	✓						Indirect
	Only focused on the ambiguities of the knowledge of innovation							
	Wang et. al. [31]	✓	✓	✓		✓	✓	Balanced Score Card
	Does not consider the status of innovative activities in the organization’s strategy							
	Herfert et. al. [32]			✓		✓		Risk level
	Only business and portfolio management tools are offered to evaluate activities against corporate strategy.							
	Rhéaume et. al. [33]			✓	✓			Indirect
	No guidelines for translating innovation strategies into resource allocation							
	Ansoff et. al. [34]	✓	✓	✓	✓	✓	✓	Synergy
	Does not issue a clear Yes/No verdict.							
	Current Research	✓	✓	✓	✓	✓	✓	Adjustable Fuzzy-DEA
	The dynamic status of activities is not considered							

To address the above gap, this study suggests using expert elicitation, as well as looking at the corporate strategy through the lens of efficiency and risk. Accordingly, the fuzzy front-end concept and adjustable fuzzy data envelopment analysis model are applied as a systematic approach to performance assessment of the innovation strategies for new product development.

## III. Adjustable fuzzy dea approach

Performance evaluation is one of the most essential real-world decision-making problems. Data envelopment analysis (DEA) is one of the most applicable and popular approaches for performance appraisal, benchmarking, and ranking the peer decision-making units (DMUs) in the presence of multiple inputs and outputs [35–37]. One of the main challenges in applying the traditional DEA approaches to real-life problems is the presence of ambiguity and uncertainty in inputs and/or outputs [38–40]. As a result, using new uncertain DEA models that can measure the performance of DMUs under an uncertain environment seems to be essential.

Accordingly, an adjustable fuzzy data envelopment analysis (AFDEA) model based on the general fuzzy measure will be introduced. The AFDEA can be applied for performance evaluation of peer homogeneous DMUs under fuzzy data and linguistic variables. Notably, in the AFDEA model, the attitude of the decision-maker could be determined by the optimistic-pessimistic parameter. The indices, parameters, and decision variables that will be employed in the AFDEA model are as follows:

**Table:** 

*Indices*:	
*i*	*the index of inputs i* = 1,…,*I*
*j*	*the index of outputs j* = 1,…,*J*
*k*	*the index of DMUs k* = 1,…,*K*
*g*	*the index of DMU under evaluation*
***Parameters*:**	
*P*_*ig*_	*the i*^*th*^ *input of DMU under evaluation*
${\tilde{P}}_{ig}$	*the i*^*th*^ *fuzzy input of DMU under evaluation*
*Q*_*jg*_	*the j*^*th*^ *output of DMU under evaluation*
${\tilde{Q}}_{jg}$	*the j*^*th*^ *fuzzy output of DMU under evaluation*
*P*_*ik*_	*the i*^*th*^ *input of k*^*th*^ *DMU*
${\tilde{P}}_{ik}$	*the i*^*th*^ *fuzzy input of k*^*th*^ *DMU*
*Q*_*jk*_	*the j*^*th*^ *output of k*^*th*^ *DMU*
${\tilde{Q}}_{jk}$	*the j*^*th*^ *fuzzy output of k*^*th*^ *DMU*
*ω*	*the optimistic-pessimistic parameter of general fuzzy measure*
*δ*	*a confidence level for satisfying the uncertain constraint*
Δ	*a large number*
***Decision Variables*:**	
*α*_*i*_	*the weight for the i*^*th*^ *input*
*β*_*j*_	*the weight for the k*^*th*^ *output*
Φ	*a continuous variable for converting the objective function to constraint*
*ξ*	*binary variable for linearization of incompatible constraints*

Now, assume that the fuzzy inputs and outputs have a triangular fuzzy (TRF) distribution as $\tilde{P}(P^{1},P^{2},P^{3})$ and $\tilde{Q}(Q^{1},Q^{2},Q^{3})$ where *P*^1^<*P*^2^<*P*^3^ and *Q*^1^<*Q*^2^<*Q*^3^. Finally, according to Peykani et al. [41], the AFDEA model will be introduced as Model (1):

$$\mathrm{Max}\;\Phi^{\mathrm{TRF}}$$ (1)

$$S.t.\;\sum_{j=1}^{J}((\frac{\delta -\omega }{1-\omega })Q_{jg}^{1}+(\frac{1-\delta }{1-\omega })Q_{jg}^{2})\beta_{j}\geq \Phi^{\mathrm{TRF}}-\Delta \xi$$

$$\sum_{j=1}^{J}((\frac{\delta }{\omega })Q_{jg}^{2}+(\frac{\omega -\delta }{\omega })Q_{jg}^{3})\beta_{j}\geq \Phi^{\mathrm{TRF}}-\Delta (1-\xi )$$

$$\sum_{i=1}^{I}((\frac{1-\delta }{1-\omega })P_{ig}^{2}+(\frac{\delta -\omega }{1-\omega })P_{ig}^{3})\alpha_{i}\leq 1+\Delta \xi$$

$$\sum_{i=1}^{I}((\frac{\omega -\delta }{\omega })P_{ig}^{1}+(\frac{\delta }{\omega })P_{ig}^{2})\alpha_{i}\leq 1+\Delta (1-\xi )$$

$$\sum_{j=1}^{J}((\frac{1-\delta }{1-\omega })Q_{jk}^{2}+(\frac{\delta -\omega }{1-\omega })Q_{jk}^{3})\beta_{j}-\sum_{i=1}^{I}((\frac{\delta -\omega }{1-\omega })P_{ik}^{1}+(\frac{1-\delta }{1-\omega })P_{ik}^{2})\alpha_{i}\leq \Delta \xi ,\;\forall k$$

$$\sum_{j=1}^{J}((\frac{\omega -\delta }{\omega })Q_{jk}^{1}+(\frac{\delta }{\omega })Q_{jk}^{2})\beta_{j}-\sum_{i=1}^{I}((\frac{\delta }{\omega })P_{ik}^{2}+(\frac{\omega -\delta }{\omega })P_{ik}^{3})\alpha_{i}\leq \Delta \left(1-\xi \right),\;\forall k$$

δ>ω−Δξ

δ≤ω+Δ(1−ξ)

ξ∈{0,1}

$$\alpha_{i},\beta_{j}\geq 0,\;\forall i,j$$

Also, assume that the fuzzy inputs and outputs have a trapezoidal fuzzy (TLF) distribution as $\tilde{P}(P^{1},P^{2},P^{3},P^{4})$ and $\tilde{Q}(Q^{1},Q^{2},Q^{3},Q^{4})$ where *P*^1^<*P*^2^<*P*^3^<*P*^4^ and *Q*^1^<*Q*^2^<*Q*^3^<*Q*^4^. Finally, the AFDEA model will be presented as Model (2):

$$\mathrm{Max}\;\Phi^{\mathrm{TLF}}$$ (2)

$$S.t.\;\sum_{j=1}^{J}((\frac{\delta -\omega }{1-\omega })Q_{jg}^{1}+(\frac{1-\delta }{1-\omega })Q_{jg}^{2})\beta_{j}\geq \Phi^{\mathrm{TLF}}-\Delta \xi$$

$$\sum_{j=1}^{J}((\frac{\delta }{\omega })Q_{jg}^{3}+(\frac{\omega -\delta }{\omega })Q_{jg}^{4})\beta_{j}\geq \Phi^{\mathrm{TLF}}-\Delta \left(1-\xi \right)$$

$$\sum_{i=1}^{I}((\frac{1-\delta }{1-\omega })P_{ig}^{3}+(\frac{\delta -\omega }{1-\omega })P_{ig}^{4})\alpha_{i}\leq 1+\Delta \xi$$

$$\sum_{i=1}^{I}((\frac{\omega -\delta }{\omega })P_{ig}^{1}+(\frac{\delta }{\omega })P_{ig}^{2})\alpha_{i}\leq 1+\Delta \left(1-\xi \right)$$

$$\sum_{j=1}^{J}((\frac{1-\delta }{1-\omega })Q_{jk}^{3}+(\frac{\delta -\omega }{1-\omega })Q_{jk}^{4})\beta_{j}-\sum_{i=1}^{I}((\frac{\delta -\omega }{1-\omega })P_{ik}^{1}+(\frac{1-\delta }{1-\omega })P_{ik}^{2})\alpha_{i}\leq \Delta \xi ,\;\forall k$$

$$\sum_{j=1}^{J}((\frac{\omega -\delta }{\omega })Q_{jk}^{1}+(\frac{\delta }{\omega })Q_{jk}^{2})\beta_{j}-\sum_{i=1}^{I}((\frac{\delta }{\omega })P_{ik}^{3}+(\frac{\omega -\delta }{\omega })P_{ik}^{4})\alpha_{i}\leq \Delta \left(1-\xi \right),\;\forall k$$

δ>ω−Δξ

δ≤ω+Δ(1−ξ)

ξ∈{0,1}

$$\alpha_{i},\beta_{j}\geq 0,\forall i,\;j$$

It should be noted that in the above models, Δ = 0, Δ = 1, and Δ = 0.5 show the pessimistic, optimistic, and compromise attitudes of the DM, respectively. In other words, the adjustable fuzzy DEA model is able to cover all previous fuzzy chance-constrained DEA models that presented based on necessity (pessimistic attitude), possibility (optimistic attitude), and credibility (compromise attitude) measures in fuzzy DEA field. Since the discriminatory power of the AFDEA model is more than the traditional DEA model, the AFDEA model can be used to fully rank the DMUs under a fuzzy environment. In this way, we can use AFDEA to assess system performance in the fuzzy front-end from a variety of optimistic as well as pessimistic perspectives.

## IV. Methodology

To model the process of an innovative project required human resources in terms of man-hour, fixed costs such as equipment, software licenses, and laboratory costs, and current costs are considered as project inputs and the outputs are considered as created value and probability of project success. Due to the nature of research and development, the assumption is that, the output of a project is a value that results from the outcomes of innovative activities.

**Fig 1:**
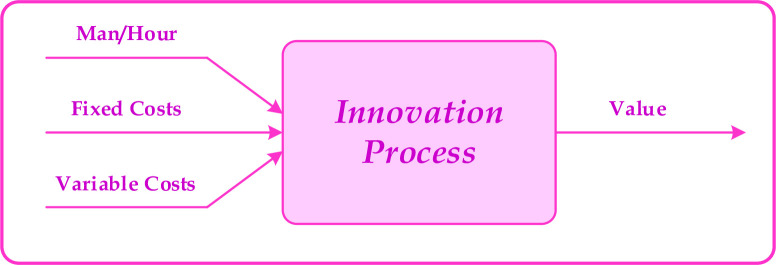
Product development process model.

Items such as production-ready prototypes as well as deposition of knowledge and technology in organization boost the created-value and items such as discredit and lost opportunity are considered as counter-value. For example, if the project succeeds, the output may be a combination of a patent and a product to be successfully marketed or, in the event of project failure, the value of knowledge applicable for other activities and projects, taking into account the counter-value of failure discredit, deduced from the organization. The model is shown in Fig 1 and presumes all inputs and outputs as fuzzy variables.

Decision-making at the go-to development gate maximizes the innovation efficiency under uncertainty, while the information is mostly vague, verbal, approximate, and unofficial. To overcome this issue, we choose fuzzy data development analysis as a part of the proposed decision support framework.

When a decision-maker attempts to decide whether to include a project within a portfolio of projects or not, he/she certainly thinks about the risks and uncertainties that may affect the expected outcome of that project. Knowledge is known as a major contribution to research on projects and their developments. There is a major difference between innovative projects and other types of projects. According to research by Shuang and his colleagues [42], knowledge is the major perspective of product development project selection. Other perspectives include economics, technology, operation, strategy, customer, partner, and resources. Knowledge contribution of innovative projects can be categorized into three criteria [43–45]:

- Individual learning.
- Organizational level learning.
- Organizational culture change.

These categories contribute to the whole organization and contrary to other project perspectives, they remain as a positive contribution even if the project fails. This influence is much higher in the case of the project’s successes. this means that innovative projects still bring value to organizations even if they fail and we should consider this “failure value” in the project selection scenario.

As already mentioned, this model assumes that a research and development project can be valuable even if it fails, as the accumulated knowledge is the last thing that the failed project brings to the organization. Generally, the value of a project in case of failure is much less than its value if the project succeeds. Hence, for the calculation of created value, the following three parameters are specified:

- Projections of project success and fail
- Prediction of created value If successful
- The estimated value created in case of failure of the project

Therefore, the created value is calculated as Eq (2):

$$V=(P_{s}\times V_{s})+(P_{f}\times V_{f})$$ (3)

In this formula, *V* is the total created value, *P*_*s*_ is the possibility of success and *P*_*f*_ is the possibility of failure, *V*_*f*_ and, is the value created in the event of success *V*_*s*_ is the value created in the event of failure.

To collect data for analysis, we consider research and development as a widely accepted and well-established innovation process within various industries. This makes the results of data analysis easier to digest by practitioners as well as researchers.

Different factors add certain kinds of uncertainty into R&D project making, so each project is a unique experience. Hence gathering probabilistic information about R&D projects is a challenge. To generate stochastic input for our decision model, we invite experts with special knowledge to express their ideas about the likelihood of values or events in question.

## V. Empirical results

The data was compiled from an R&D department at a large IT company that has a long history of developing technologies for data communication. According to Namazi [46], this R&D unit has been a role model within the country, winning international innovation awards. Consequently, this unit can be regarded as a representative at the national and international levels. It can be seen from the history of this R&D unit that it comes out every 4 years with an updated platform and a range of main and by-products. At the time of data collection, the R&D center was planning for the next 5 years, in which 33 projects were proposed for implementation. Additionally, the unit employed 40 knowledge-based employees, including 10 senior researchers and engineers who, in addition to technical knowledge, had a thorough understanding of the product market.

Hence, 33 projects were selected from a research and development department, then 10 senior experts with research and development, marketing, and product management history were selected to elaborate on their opinions about those projects.

Experts were asked to give their opinions based on their experience and understanding of the level of complexity, technology, required knowledge, future risks, and market needs regarding each project. They also were asked to discuss the possibility of combined technical and marketing success or failure of projects, the expected value in the event of success or failure of the project in addition to estimates of required resources such as man-hours, fixed costs, and research and development costs.

**Fig 2:**
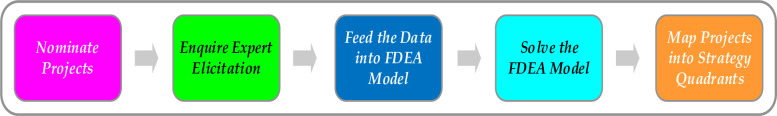
Research process.

After data collection, we will feed the data into the FDEA model and then map the analysis results into the proposed strategy map. The above-mentioned process is summarized in Fig 2.

We insist that we have asked out an expert about the possibility of success and failure in the realm of fuzzy theory. As they were fully aware of fuzzy theory concepts, they simply gave their opinions about possibilities on a fuzzy natural language expression as illustrated in Table 3.

**Table 3:** Verbal fuzzy variables.

*Linguistic Variables*	*Possibility of Success*		
	Low	Mid	High
*Extremely possible*	70	90	100
*Possible*	60	80	90
*Almost Possible*	30	50	70
*Hardly Possible*	10	20	40
*Not Possible*	0	10	30

Experts were asked to retrieve their responses in a fuzzy way to collect the fuzzy data. According to Table 3 triangular verbal fuzzy variables were used to reflect the probability of project success. In addition, regarding the value of success and failure value, the mean of opinions was considered as a fuzzy norm (triangular fuzzy midpoint), and minimum and maximum values were given as upper bound and lower bound of a fuzzy number, respectively. The Project Planning Unit was also asked to provide triangular fuzzy numbers for the project’s required resources. The results are shown in Tables 4 and 5. It is imperative to note that according to Eq (3), the outputs will eventually be merged to produce a single output to calculate performance.

**Table 4:** Fuzzy inputs.

*Projects*	*Man-Hour*			*Fixed Costs*			*Current Costs*		
	Min	Mid	Max	Min	Mid	Max	Min	Mid	Max
*P1*	32	40	48	240	300	360	80	100	120
*P2*	24	30	36	160	200	240	120	150	180
*P3*	0.16	0.2	0.24	1.6	2	2.4	0.8	1	1.2
*P4*	8	10	12	20	25	30	1.6	2	2.4
*P5*	4	5	6	0.8	1	1.2	1.6	2	2.4
*P6*	8	10	12	4	5	6	8	10	12
*P7*	16	20	24	3.2	4	4.8	8	10	12
*P8*	2.4	3	3.6	40	50	60	16	20	24
*P9*	12	15	18	1.6	2	2.4	1.6	2	2.4
*P10*	1.6	2	2.4	1.6	2	2.4	4	5	6
*P11*	4	5	6	1.6	2	2.4	0.8	1	1.2
*P12*	8	10	12	20	25	30	1.6	2	2.4
*P13*	8	10	12	4	5	6	5.6	7	8.4
*P14*	8	10	12	3.2	4	4.8	0.8	1	1.2
*P15*	4	5	6	0.8	1	1.2	0.8	1	1.2
*P16*	8	10	12	1.6	2	2.4	3.2	4	4.8
*P17*	3.2	4	4.8	0.8	1	1.2	0.8	1	1.2
*P18*	2.4	3	3.6	0.4	0.5	0.6	0.8	1	1.2
*P19*	4	5	6	8	10	12	4	5	6
*P20*	3.2	4	4.8	4	5	6	0.16	0.2	0.24
*P21*	1.6	2	2.4	40	50	60	16	20	24
*P22*	0.8	1	1.2	1.6	2	2.4	0.4	0.5	0.6
*P23*	0.8	1	1.2	0.8	1	1.2	4	5	6
*P24*	1.6	2	2.4	0.8	1	1.2	0.8	1	1.2
*P25*	0.4	0.5	0.6	0.16	0.2	0.24	0.8	1	1.2
*P26*	0.4	0.5	0.6	0.4	0.5	0.6	0.4	0.5	0.6
*P27*	1.6	2	2.4	40	50	60	16	20	24
*P28*	0.4	0.5	0.6	0.16	0.2	0.24	0.8	1	1.2
*P29*	0.16	0.2	0.24	0.08	0.1	0.12	0.16	0.2	0.24
*P30*	0.8	1	1.2	0.8	1	1.2	0.4	0.5	0.6
*P31*	0.16	0.2	0.24	1.6	2	2.4	0.8	1	1.2
*P32*	0.4	0.5	0.6	0.16	0.2	0.24	0.8	1	1.2
*P33*	0.4	0.5	0.6	0.16	0.2	0.24	0.8	1	1.2

**Table 5:** Fuzzy outputs.

*Projects*	*Possibility of Success*			*Success Value*			*Possibility of Fail*			*Fail Value*		
	Min	Mid	Max	Min	Mid	Max	Min	Mid	Max	Min	Mid	Max
*P1*	30	50	70	80	97	100	30	50	70	16	33	45
*P2*	60	80	90	50	79	100	10	20	40	5	24	40
*P3*	70	90	100	40	59	80	0	10	30	1	13	24
*P4*	70	90	100	60	73	80	0	10	30	1	14	24
*P5*	60	80	90	40	60	90	10	20	40	2	11	27
*P6*	30	50	70	30	56	100	30	50	70	2	9	25
*P7*	70	90	100	60	87	100	0	10	30	6	15	40
*P8*	60	80	90	30	53	70	10	20	40	2	10	21
*P9*	70	90	100	30	70	100	0	10	30	2	8	30
*P10*	60	80	90	20	37	50	10	20	40	1	5	10
*P11*	30	50	70	30	40	60	30	50	70	1	4	9
*P12*	70	90	100	30	57	80	0	10	30	1	7	24
*P13*	60	80	90	30	64	80	10	20	40	2	7	12
*P14*	60	80	90	20	53	80	10	20	40	1	7	20
*P15*	60	80	90	20	54	80	10	20	40	1	9	20
*P16*	30	50	70	20	50	80	30	50	70	1	5	8
*P17*	70	90	100	40	83	100	0	10	30	1	9	20
*P18*	60	80	90	30	41	60	10	20	40	1	4	9
*P19*	10	20	40	30	43	70	60	80	90	1	2	7
*P20*	70	90	100	30	43	70	0	10	30	2	5	14
*P21*	60	80	90	10	34	40	10	20	40	1	2	4
*P22*	30	50	70	30	51	80	30	50	70	1	2	4
*P23*	60	80	90	10	30	50	10	20	40	1	1	3
*P24*	70	90	100	20	37	50	0	10	30	1	3	5
*P25*	70	90	100	30	36	40	0	10	30	1	2	4
*P26*	70	90	100	20	33	50	0	10	30	1	1	3
*P27*	60	80	90	10	27	50	10	20	40	1	2	3
*P28*	70	90	100	10	23	30	0	10	30	1	1	2
*P29*	70	90	100	10	20	30	0	10	30	1	1	2
*P30*	70	90	100	10	30	50	0	10	30	1	2	5
*P31*	60	80	90	10	33	60	10	20	40	1	2	6
*P32*	70	90	100	10	23	40	0	10	30	1	1	2
*P33*	70	90	100	10	28	50	0	10	30	1	1	3

Now, using adjustable fuzzy data envelopment analysis, we calculate the efficiency of projects in two ways:

1. Pessimistic: By selecting the required measure and setting the parameter to a 100% confidence level where the model constraints are maximally met.
2. Optimistic: Selecting a measure of probability and set the confidence level parameter to 0% which means that the model constraints are kept at a minimum level.

The model output is shown in Table 6. It is natural to calculate the lowest performance in the skeptical state and the highest performance in the optimistic one for the projects. The full results of calculations are presented in Appendixes A to C in S1 Appendix.

**Table 6:** Projects efficiencies in optimistic and pessimistic fuzzy approaches.

*Projects*	*Necessity (Confidence Level = 100%)*	*Possibility (Confidence Level = 0%)*
*P1*	0.001852	0.051176
*P2*	0.002667	0.050013
*P3*	0.014028	4.671429
*P4*	0.014917	1.314921
*P5*	0.012024	1.967143
*P6*	0.004107	0.375000
*P7*	0.012181	0.553846
*P8*	0.012024	0.255000
*P9*	0.008450	2.244118
*P10*	0.012024	0.763636
*P11*	0.006012	1.844182
*P12*	0.010898	1.314921
*P13*	0.008562	0.462120
*P14*	0.009478	2.666667
*P15*	0.012024	3.294118
*P16*	0.004525	0.660000
*P17*	0.014028	4.364706
*P18*	0.012024	2.468571
*P19*	0.002004	0.294000
*P20*	0.014028	5.300000
*P21*	0.012024	0.201429
*P22*	0.006012	4.560000
*P23*	0.012024	1.440000
*P24*	0.014028	2.120588
*P25*	0.014028	2.942857
*P26*	0.014028	4.362857
*P27*	0.012024	0.247500
*P28*	0.014028	2.185714
*P29*	0.014028	6.557143
*P30*	0.014028	3.993878
*P31*	0.012024	3.021429
*P32*	0.014028	2.900000
*P33*	0.014028	3.635714

**Fig 3:**
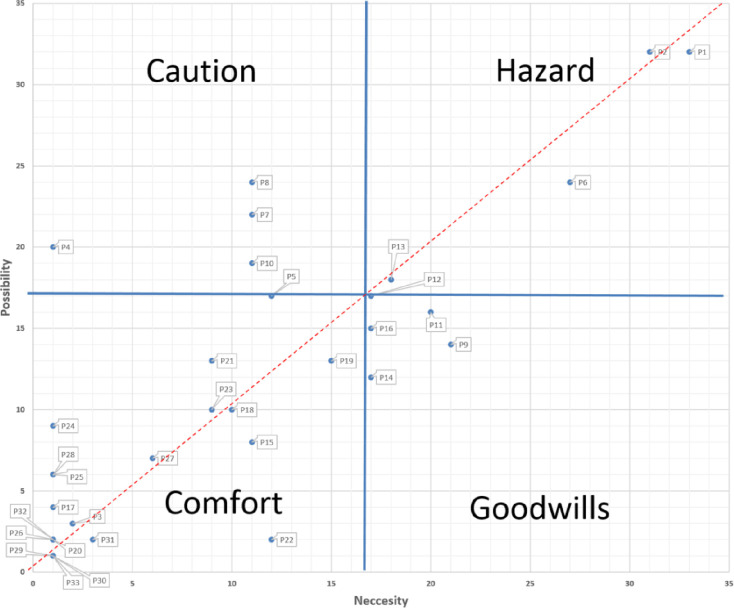
Project performance strategy map with two feasibility measures.

It may be argued here that credit rating can be used to make decisions. Credit rating, the average of the two measures, seems not to be appropriate for our decision-making because it ignores the gap between the possibility and the requirement of much awareness of the two perspectives. On the contrary, the validity view assumes the importance of the two possibilities for the same decision-maker if this assumption is not necessarily true. As a result, the credit perspective may lead us astray in decision-making. For this reason, we combined performance-based performance diagrams with the requirement of validity. As shown in Fig 3, the blue lines are the average efficient rank of 17, which creates four regions or quadrants:

- Comfort Zone (Bottom-Left): These projects perform fairly well in both pessimistic and optimistic approaches.
- Good Wills Zone (Bottom-Right): These projects only work well in an optimistic approach.
- Caution Zone (Top-Left): These projects only work well in a pessimistic sense.
- Hazard Zone (Top-Right): These projects perform relatively low in both optimistic and pessimistic approaches.

In addition, projects under the red dotted line will perform better in pessimistic conditions, while projects over the red line will perform better under optimistic conditions. Projects 1 and 2 are in the "hazard zone" and Project 9 is in the "good faith zone" which means no matter how hard or easy it is, the performance of Projects 1 and 2 is not good, and Project 9 works well only in optimistic conditions. Individual Projects 5 and 19 are located at the border of "comfort zone", "caution zone" and "comfort zone", respectively. We can hope Project 5 has a moderate efficiency in an optimistic approach. The status of Project 19 is only slightly better because it will have average performance in both cases. Projects 3 and 17 are good choices, while optimism is needed for Project 7. Projects 8, 27, and 21 need optimism to be added to the portfolio. Project 4 can also be a good option if optimism is concerned, while Project 12 is clearly at the center of the chart and no definitive judgment can be made. Project 31 can be a good option as it is efficient and performs well in both approaches. Project 13 is in the “hazard zone” near the center and Project 6 is also in that area, so we don’t have much hope for the decision-maker to put them in the portfolio unless there is a specific reason. Likewise, decisions about other projects can be easily made.

## VI. Summary and conclusions

Technology managers are always struggling to select projects and ideas for their team and they are faced with a high level of uncertainty while evaluating projects. This paper presents a method to evaluate uncertain innovative activities using expert elicitation on resources and outcomes of projects. The hierarchical structure that governs large corporations makes the senior executives’ perception of technology influenced by intra-organizational political behavior [47], and this affects the technology decision-making process. When technological innovation is at the forefront of a company’s growth, the company’s survival depends heavily on the quality of decisions about technology.

However, as stated before, decision-making in the early stages of innovation should not be based on political or individual power, but on the intuition and consensus of decision-makers. This paper showed that these innovative activities mainly have low efficiency and high uncertainty, and the decision to implement or abandon them requires reviewing and balancing the goals and strategic approach of the organization with technological and business features. According to the above introduction, the following practical recommendations are given for the attention of respected managers:

- The strategic position of innovative activities can be determined by focusing on efficiency and uncertainty at the same time.
- The decision-making team should consider multi-purposes as much as possible and take advantage of the participation of different experts and stakeholders.
- Use both optimistic and pessimistic views to balance the decisions.
- The use of the intuition of those who are going to develop the product/technology would greatly improve the decision-making process. These people are usually buried under the organizational hierarchy. Developing a strategic discourse at all levels of the organization will enhance the quality of this partnership.
- Providing a clear definition of the acceptable risk would ensure that all decision-makers have a common understanding of this definition.

This research has practically introduced technical knowledge and market awareness into decision-making by surveying experts and gaining their opinions about the value and probability of success of projects. Moreover, it could provide a solution for risk management of innovative activities in the form of a portfolio of projects with differentiated risks and efficiency. Hence, as seen in Table 2, the results of this study are only comparable to the research by Ansoff et. al. [34] as it has covered all aspects previously focused on by researchers. In contrast to previous work, this study provides a solution for strategic project selection as well as considering the technology development team’s intuition from both optimistic and pessimistic perspectives. The research focuses only on the fuzzy front-end of the innovation process, which is a limitation. Similarly, this research did not examine the relationship between projects and their dynamic status.

For future research directions, the Z-number theory can be applied to deal with data ambiguity and increase the reliability of expert opinions. Additionally, other fuzzy concepts such as fuzzy type-2 and random fuzzy variables can be utilized to present the new version of the fuzzy DEA approach. Furthermore, the scenario-based robust optimization approach can be applied for considering different possible scenarios for new product development.

## Supporting information

S1 Appendix (DOCX)

S1 File (DOCX)
