# Supplementary material for: Bridging the knowledge gap between technology and business: An innovation strategy perspective
Source: PLoS One. 2022 Apr 14;17(4):e0266843. doi: 10.1371/journal.pone.0266843 (PMC9009678; doi:10.1371/journal.pone.0266843)
Supplement: S1 Appendix — (DOCX) [file pone.0266843.s001.docx]

**APPENDIX A. The Results under Necessity Measure**

| Projects |  | Confidence Level | | | | | | | | |
| --- | --- | --- | --- | --- | --- | --- | --- | --- | --- | --- |
|  |  | 0% |  | 25% |  | 50% |  | 75% |  | 100% |
| P1 |  | 0.010290 |  | 0.006924 |  | 0.004609 |  | 0.002994 |  | 0.001852 |
| P2 |  | 0.010876 |  | 0.007360 |  | 0.004927 |  | 0.003286 |  | 0.002667 |
| P3 |  | 0.999266 |  | 0.106710 |  | 0.040974 |  | 0.021878 |  | 0.014028 |
| P4 |  | 0.296992 |  | 0.104143 |  | 0.048124 |  | 0.025221 |  | 0.014917 |
| P5 |  | 0.276902 |  | 0.095876 |  | 0.036538 |  | 0.018961 |  | 0.012024 |
| P6 |  | 0.035693 |  | 0.022769 |  | 0.014113 |  | 0.008076 |  | 0.004107 |
| P7 |  | 0.110228 |  | 0.062751 |  | 0.034948 |  | 0.020161 |  | 0.012181 |
| P8 |  | 0.059214 |  | 0.048935 |  | 0.033425 |  | 0.018961 |  | 0.012024 |
| P9 |  | 0.343735 |  | 0.081897 |  | 0.034193 |  | 0.016262 |  | 0.008450 |
| P10 |  | 0.153368 |  | 0.074971 |  | 0.033410 |  | 0.018961 |  | 0.012024 |
| P11 |  | 0.226608 |  | 0.042311 |  | 0.018092 |  | 0.010210 |  | 0.006012 |
| P12 |  | 0.231129 |  | 0.076069 |  | 0.032474 |  | 0.017140 |  | 0.010898 |
| P13 |  | 0.082413 |  | 0.052971 |  | 0.028316 |  | 0.014736 |  | 0.008562 |
| P14 |  | 0.413433 |  | 0.064518 |  | 0.025841 |  | 0.013270 |  | 0.009478 |
| P15 |  | 0.487341 |  | 0.083122 |  | 0.032253 |  | 0.018961 |  | 0.012024 |
| P16 |  | 0.075825 |  | 0.033958 |  | 0.014982 |  | 0.007051 |  | 0.004525 |
| P17 |  | 0.813252 |  | 0.142883 |  | 0.048076 |  | 0.022895 |  | 0.014028 |
| P18 |  | 0.374444 |  | 0.075445 |  | 0.033410 |  | 0.018961 |  | 0.012024 |
| P19 |  | 0.022436 |  | 0.014559 |  | 0.007002 |  | 0.003647 |  | 0.002004 |
| P20 |  | 0.999266 |  | 0.081041 |  | 0.037329 |  | 0.021878 |  | 0.014028 |
| P21 |  | 0.088821 |  | 0.073402 |  | 0.033410 |  | 0.018961 |  | 0.012024 |
| P22 |  | 0.555370 |  | 0.052378 |  | 0.019194 |  | 0.010210 |  | 0.006012 |
| P23 |  | 0.241679 |  | 0.078540 |  | 0.033410 |  | 0.018961 |  | 0.012024 |
| P24 |  | 0.363485 |  | 0.087375 |  | 0.038182 |  | 0.021878 |  | 0.014028 |
| P25 |  | 0.892082 |  | 0.089010 |  | 0.038182 |  | 0.021878 |  | 0.014028 |
| P26 |  | 0.654629 |  | 0.089010 |  | 0.038182 |  | 0.021878 |  | 0.014028 |
| P27 |  | 0.088821 |  | 0.073402 |  | 0.033410 |  | 0.018961 |  | 0.012024 |
| P28 |  | 0.569994 |  | 0.089010 |  | 0.038182 |  | 0.021878 |  | 0.014028 |
| P29 |  | 0.999266 |  | 0.089010 |  | 0.038182 |  | 0.021878 |  | 0.014028 |
| P30 |  | 0.565507 |  | 0.089010 |  | 0.038182 |  | 0.021878 |  | 0.014028 |
| P31 |  | 0.888212 |  | 0.078540 |  | 0.033410 |  | 0.018961 |  | 0.012024 |
| P32 |  | 0.569994 |  | 0.089010 |  | 0.038182 |  | 0.021878 |  | 0.014028 |
| P33 |  | 0.705897 |  | 0.089010 |  | 0.038182 |  | 0.021878 |  | 0.014028 |

**APPENDIX B. The Results under Credibility Measure**

| Projects |  | Confidence Level | | | | | | | | |
| --- | --- | --- | --- | --- | --- | --- | --- | --- | --- | --- |
|  |  | 0% |  | 25% |  | 50% |  | 75% |  | 100% |
| P1 |  | 0.051176 |  | 0.021919 |  | 0.010307 |  | 0.004605 |  | 0.001852 |
| P2 |  | 0.050013 |  | 0.022807 |  | 0.010893 |  | 0.004923 |  | 0.002667 |
| P3 |  | 4.671429 |  | 2.104499 |  | 1.000000 |  | 0.688995 |  | 0.014028 |
| P4 |  | 1.314921 |  | 0.580461 |  | 0.297450 |  | 0.143124 |  | 0.014917 |
| P5 |  | 1.967143 |  | 0.691456 |  | 0.277348 |  | 0.124995 |  | 0.012024 |
| P6 |  | 0.375000 |  | 0.116730 |  | 0.035757 |  | 0.014099 |  | 0.004107 |
| P7 |  | 0.553846 |  | 0.231236 |  | 0.110401 |  | 0.051212 |  | 0.012181 |
| P8 |  | 0.255000 |  | 0.114944 |  | 0.059259 |  | 0.040191 |  | 0.012024 |
| P9 |  | 2.244118 |  | 0.818171 |  | 0.344345 |  | 0.138714 |  | 0.008450 |
| P10 |  | 0.763636 |  | 0.346761 |  | 0.153627 |  | 0.065386 |  | 0.012024 |
| P11 |  | 1.844182 |  | 0.630830 |  | 0.226993 |  | 0.097274 |  | 0.006012 |
| P12 |  | 1.314921 |  | 0.524588 |  | 0.231537 |  | 0.096051 |  | 0.010898 |
| P13 |  | 0.462120 |  | 0.178178 |  | 0.082557 |  | 0.033704 |  | 0.008562 |
| P14 |  | 2.666667 |  | 1.004639 |  | 0.414207 |  | 0.157607 |  | 0.009478 |
| P15 |  | 3.294118 |  | 1.185875 |  | 0.488235 |  | 0.187559 |  | 0.012024 |
| P16 |  | 0.660000 |  | 0.216932 |  | 0.075967 |  | 0.028393 |  | 0.004525 |
| P17 |  | 4.364706 |  | 1.718443 |  | 0.814661 |  | 0.338509 |  | 0.014028 |
| P18 |  | 2.468571 |  | 0.891492 |  | 0.375028 |  | 0.174859 |  | 0.012024 |
| P19 |  | 0.294000 |  | 0.086617 |  | 0.022475 |  | 0.009254 |  | 0.002004 |
| P20 |  | 5.300000 |  | 2.304663 |  | 1.000000 |  | 0.688995 |  | 0.014028 |
| P21 |  | 0.201429 |  | 0.129861 |  | 0.088889 |  | 0.060287 |  | 0.012024 |
| P22 |  | 4.560000 |  | 1.551747 |  | 0.556352 |  | 0.226208 |  | 0.006012 |
| P23 |  | 1.440000 |  | 0.612519 |  | 0.242121 |  | 0.120574 |  | 0.012024 |
| P24 |  | 2.120588 |  | 0.807092 |  | 0.364098 |  | 0.156152 |  | 0.014028 |
| P25 |  | 2.942857 |  | 1.685081 |  | 0.893370 |  | 0.448068 |  | 0.014028 |
| P26 |  | 4.362857 |  | 1.569314 |  | 0.655691 |  | 0.293470 |  | 0.014028 |
| P27 |  | 0.247500 |  | 0.129861 |  | 0.088889 |  | 0.060287 |  | 0.012024 |
| P28 |  | 2.185714 |  | 1.190168 |  | 0.570995 |  | 0.344498 |  | 0.014028 |
| P29 |  | 6.557143 |  | 2.371403 |  | 1.000000 |  | 0.688995 |  | 0.014028 |
| P30 |  | 3.993878 |  | 1.428166 |  | 0.566558 |  | 0.275598 |  | 0.014028 |
| P31 |  | 3.021429 |  | 1.298611 |  | 0.888889 |  | 0.602871 |  | 0.012024 |
| P32 |  | 2.900000 |  | 1.406567 |  | 0.570995 |  | 0.344498 |  | 0.014028 |
| P33 |  | 3.635714 |  | 1.752906 |  | 0.707182 |  | 0.344498 |  | 0.014028 |

**APPENDIX C. The Results under Possibility Measure**

| Projects |  | Confidence Level | | | | | | | | |
| --- | --- | --- | --- | --- | --- | --- | --- | --- | --- | --- |
|  |  | 0% |  | 25% |  | 50% |  | 75% |  | 100% |
| P1 |  | 0.051176 |  | 0.032799 |  | 0.021902 |  | 0.014948 |  | 0.010322 |
| P2 |  | 0.050013 |  | 0.033651 |  | 0.022790 |  | 0.015699 |  | 0.010909 |
| P3 |  | 4.671429 |  | 3.092766 |  | 2.102911 |  | 1.447974 |  | 1.001499 |
| P4 |  | 1.314921 |  | 0.848707 |  | 0.580043 |  | 0.410521 |  | 0.297825 |
| P5 |  | 1.967143 |  | 1.126064 |  | 0.690808 |  | 0.436463 |  | 0.277860 |
| P6 |  | 0.375000 |  | 0.203938 |  | 0.116601 |  | 0.066445 |  | 0.035854 |
| P7 |  | 0.553846 |  | 0.346971 |  | 0.231058 |  | 0.158396 |  | 0.110557 |
| P8 |  | 0.255000 |  | 0.168869 |  | 0.114858 |  | 0.079118 |  | 0.059304 |
| P9 |  | 2.244118 |  | 1.308569 |  | 0.817435 |  | 0.527430 |  | 0.344936 |
| P10 |  | 0.763636 |  | 0.541596 |  | 0.346464 |  | 0.228859 |  | 0.153872 |
| P11 |  | 1.844182 |  | 1.050797 |  | 0.630199 |  | 0.382192 |  | 0.227491 |
| P12 |  | 1.314921 |  | 0.810334 |  | 0.524146 |  | 0.347009 |  | 0.231916 |
| P13 |  | 0.462120 |  | 0.275991 |  | 0.178031 |  | 0.119757 |  | 0.082678 |
| P14 |  | 2.666667 |  | 1.595281 |  | 1.003737 |  | 0.644596 |  | 0.414956 |
| P15 |  | 3.294118 |  | 1.910137 |  | 1.184789 |  | 0.757375 |  | 0.489104 |
| P16 |  | 0.660000 |  | 0.367100 |  | 0.216709 |  | 0.129707 |  | 0.076138 |
| P17 |  | 4.364706 |  | 2.635118 |  | 1.717058 |  | 1.167469 |  | 0.815813 |
| P18 |  | 2.468571 |  | 1.430010 |  | 0.890687 |  | 0.574140 |  | 0.375670 |
| P19 |  | 0.294000 |  | 0.156404 |  | 0.086514 |  | 0.046650 |  | 0.022551 |
| P20 |  | 5.300000 |  | 3.467009 |  | 2.302788 |  | 1.528798 |  | 1.001765 |
| P21 |  | 0.201429 |  | 0.157812 |  | 0.129811 |  | 0.107215 |  | 0.088955 |
| P22 |  | 4.560000 |  | 2.590169 |  | 1.550190 |  | 0.938489 |  | 0.557577 |
| P23 |  | 1.440000 |  | 0.992278 |  | 0.611945 |  | 0.385232 |  | 0.242583 |
| P24 |  | 2.120588 |  | 1.260579 |  | 0.806409 |  | 0.536229 |  | 0.364658 |
| P25 |  | 2.942857 |  | 2.212282 |  | 1.684179 |  | 1.244789 |  | 0.894522 |
| P26 |  | 4.362857 |  | 2.522883 |  | 1.567889 |  | 1.007742 |  | 0.656826 |
| P27 |  | 0.247500 |  | 0.157812 |  | 0.129811 |  | 0.107215 |  | 0.088955 |
| P28 |  | 2.185714 |  | 1.620348 |  | 1.189409 |  | 0.833924 |  | 0.571849 |
| P29 |  | 6.557143 |  | 3.800866 |  | 2.369266 |  | 1.528812 |  | 1.001707 |
| P30 |  | 3.993878 |  | 2.317194 |  | 1.426828 |  | 0.899238 |  | 0.567631 |
| P31 |  | 3.021429 |  | 1.919501 |  | 1.298110 |  | 1.072146 |  | 0.889554 |
| P32 |  | 2.900000 |  | 2.021018 |  | 1.405546 |  | 0.923729 |  | 0.572126 |
| P33 |  | 3.635714 |  | 2.529282 |  | 1.751599 |  | 1.149584 |  | 0.708600 |
