# Supplementary material for: Bridging the knowledge gap between technology and business: An innovation strategy perspective
Source: PLoS One. 2022 Apr 14;17(4):e0266843. doi: 10.1371/journal.pone.0266843 (PMC9009678; doi:10.1371/journal.pone.0266843)
Supplement: S1 File — (DOCX) [file pone.0266843.s002.docx]

**Real-World Data from Iranian Industry (R&D Projects) - Fuzzy Inputs**

| Projects |  | Man-Hour | | |  | Fixed Costs | | |  | Current Costs | | |
| --- | --- | --- | --- | --- | --- | --- | --- | --- | --- | --- | --- | --- |
|  |  | Min | Mid | Max |  | Min | Mid | Max |  | Min | Mid | Max |
| P1 |  | 32 | 40 | 48 |  | 240 | 300 | 360 |  | 80 | 100 | 120 |
| P2 |  | 24 | 30 | 36 |  | 160 | 200 | 240 |  | 120 | 150 | 180 |
| P3 |  | 0.16 | 0.2 | 0.24 |  | 1.6 | 2 | 2.4 |  | 0.8 | 1 | 1.2 |
| P4 |  | 8 | 10 | 12 |  | 20 | 25 | 30 |  | 1.6 | 2 | 2.4 |
| P5 |  | 4 | 5 | 6 |  | 0.8 | 1 | 1.2 |  | 1.6 | 2 | 2.4 |
| P6 |  | 8 | 10 | 12 |  | 4 | 5 | 6 |  | 8 | 10 | 12 |
| P7 |  | 16 | 20 | 24 |  | 3.2 | 4 | 4.8 |  | 8 | 10 | 12 |
| P8 |  | 2.4 | 3 | 3.6 |  | 40 | 50 | 60 |  | 16 | 20 | 24 |
| P9 |  | 12 | 15 | 18 |  | 1.6 | 2 | 2.4 |  | 1.6 | 2 | 2.4 |
| P10 |  | 1.6 | 2 | 2.4 |  | 1.6 | 2 | 2.4 |  | 4 | 5 | 6 |
| P11 |  | 4 | 5 | 6 |  | 1.6 | 2 | 2.4 |  | 0.8 | 1 | 1.2 |
| P12 |  | 8 | 10 | 12 |  | 20 | 25 | 30 |  | 1.6 | 2 | 2.4 |
| P13 |  | 8 | 10 | 12 |  | 4 | 5 | 6 |  | 5.6 | 7 | 8.4 |
| P14 |  | 8 | 10 | 12 |  | 3.2 | 4 | 4.8 |  | 0.8 | 1 | 1.2 |
| P15 |  | 4 | 5 | 6 |  | 0.8 | 1 | 1.2 |  | 0.8 | 1 | 1.2 |
| P16 |  | 8 | 10 | 12 |  | 1.6 | 2 | 2.4 |  | 3.2 | 4 | 4.8 |
| P17 |  | 3.2 | 4 | 4.8 |  | 0.8 | 1 | 1.2 |  | 0.8 | 1 | 1.2 |
| P18 |  | 2.4 | 3 | 3.6 |  | 0.4 | 0.5 | 0.6 |  | 0.8 | 1 | 1.2 |
| P19 |  | 4 | 5 | 6 |  | 8 | 10 | 12 |  | 4 | 5 | 6 |
| P20 |  | 3.2 | 4 | 4.8 |  | 4 | 5 | 6 |  | 0.16 | 0.2 | 0.24 |
| P21 |  | 1.6 | 2 | 2.4 |  | 40 | 50 | 60 |  | 16 | 20 | 24 |
| P22 |  | 0.8 | 1 | 1.2 |  | 1.6 | 2 | 2.4 |  | 0.4 | 0.5 | 0.6 |
| P23 |  | 0.8 | 1 | 1.2 |  | 0.8 | 1 | 1.2 |  | 4 | 5 | 6 |
| P24 |  | 1.6 | 2 | 2.4 |  | 0.8 | 1 | 1.2 |  | 0.8 | 1 | 1.2 |
| P25 |  | 0.4 | 0.5 | 0.6 |  | 0.16 | 0.2 | 0.24 |  | 0.8 | 1 | 1.2 |
| P26 |  | 0.4 | 0.5 | 0.6 |  | 0.4 | 0.5 | 0.6 |  | 0.4 | 0.5 | 0.6 |
| P27 |  | 1.6 | 2 | 2.4 |  | 40 | 50 | 60 |  | 16 | 20 | 24 |
| P28 |  | 0.4 | 0.5 | 0.6 |  | 0.16 | 0.2 | 0.24 |  | 0.8 | 1 | 1.2 |
| P29 |  | 0.16 | 0.2 | 0.24 |  | 0.08 | 0.1 | 0.12 |  | 0.16 | 0.2 | 0.24 |
| P30 |  | 0.8 | 1 | 1.2 |  | 0.8 | 1 | 1.2 |  | 0.4 | 0.5 | 0.6 |
| P31 |  | 0.16 | 0.2 | 0.24 |  | 1.6 | 2 | 2.4 |  | 0.8 | 1 | 1.2 |
| P32 |  | 0.4 | 0.5 | 0.6 |  | 0.16 | 0.2 | 0.24 |  | 0.8 | 1 | 1.2 |
| P33 |  | 0.4 | 0.5 | 0.6 |  | 0.16 | 0.2 | 0.24 |  | 0.8 | 1 | 1.2 |

**Real-World Data from Iranian Industry (R&D Projects) - Fuzzy Outputs**

| Projects |  | Possibility of Success | | |  | Success Value | | |  | Possibility of Fail | | |  | Fail Value | | |
| --- | --- | --- | --- | --- | --- | --- | --- | --- | --- | --- | --- | --- | --- | --- | --- | --- |
|  |  | Min | Mid | Max |  | Min | Mid | Max |  | Min | Mid | Max |  | Min | Mid | Max |
| P1 |  | 30 | 50 | 70 |  | 80 | 97 | 100 |  | 30 | 50 | 70 |  | 16 | 33 | 45 |
| P2 |  | 60 | 80 | 90 |  | 50 | 79 | 100 |  | 10 | 20 | 40 |  | 5 | 24 | 40 |
| P3 |  | 70 | 90 | 100 |  | 40 | 59 | 80 |  | 0 | 10 | 30 |  | 1 | 13 | 24 |
| P4 |  | 70 | 90 | 100 |  | 60 | 73 | 80 |  | 0 | 10 | 30 |  | 1 | 14 | 24 |
| P5 |  | 60 | 80 | 90 |  | 40 | 60 | 90 |  | 10 | 20 | 40 |  | 2 | 11 | 27 |
| P6 |  | 30 | 50 | 70 |  | 30 | 56 | 100 |  | 30 | 50 | 70 |  | 2 | 9 | 25 |
| P7 |  | 70 | 90 | 100 |  | 60 | 87 | 100 |  | 0 | 10 | 30 |  | 6 | 15 | 40 |
| P8 |  | 60 | 80 | 90 |  | 30 | 53 | 70 |  | 10 | 20 | 40 |  | 2 | 10 | 21 |
| P9 |  | 70 | 90 | 100 |  | 30 | 70 | 100 |  | 0 | 10 | 30 |  | 2 | 8 | 30 |
| P10 |  | 60 | 80 | 90 |  | 20 | 37 | 50 |  | 10 | 20 | 40 |  | 1 | 5 | 10 |
| P11 |  | 30 | 50 | 70 |  | 30 | 40 | 60 |  | 30 | 50 | 70 |  | 1 | 4 | 9 |
| P12 |  | 70 | 90 | 100 |  | 30 | 57 | 80 |  | 0 | 10 | 30 |  | 1 | 7 | 24 |
| P13 |  | 60 | 80 | 90 |  | 30 | 64 | 80 |  | 10 | 20 | 40 |  | 2 | 7 | 12 |
| P14 |  | 60 | 80 | 90 |  | 20 | 53 | 80 |  | 10 | 20 | 40 |  | 1 | 7 | 20 |
| P15 |  | 60 | 80 | 90 |  | 20 | 54 | 80 |  | 10 | 20 | 40 |  | 1 | 9 | 20 |
| P16 |  | 30 | 50 | 70 |  | 20 | 50 | 80 |  | 30 | 50 | 70 |  | 1 | 5 | 8 |
| P17 |  | 70 | 90 | 100 |  | 40 | 83 | 100 |  | 0 | 10 | 30 |  | 1 | 9 | 20 |
| P18 |  | 60 | 80 | 90 |  | 30 | 41 | 60 |  | 10 | 20 | 40 |  | 1 | 4 | 9 |
| P19 |  | 10 | 20 | 40 |  | 30 | 43 | 70 |  | 60 | 80 | 90 |  | 1 | 2 | 7 |
| P20 |  | 70 | 90 | 100 |  | 30 | 43 | 70 |  | 0 | 10 | 30 |  | 2 | 5 | 14 |
| P21 |  | 60 | 80 | 90 |  | 10 | 34 | 40 |  | 10 | 20 | 40 |  | 1 | 2 | 4 |
| P22 |  | 30 | 50 | 70 |  | 30 | 51 | 80 |  | 30 | 50 | 70 |  | 1 | 2 | 4 |
| P23 |  | 60 | 80 | 90 |  | 10 | 30 | 50 |  | 10 | 20 | 40 |  | 1 | 1 | 3 |
| P24 |  | 70 | 90 | 100 |  | 20 | 37 | 50 |  | 0 | 10 | 30 |  | 1 | 3 | 5 |
| P25 |  | 70 | 90 | 100 |  | 30 | 36 | 40 |  | 0 | 10 | 30 |  | 1 | 2 | 4 |
| P26 |  | 70 | 90 | 100 |  | 20 | 33 | 50 |  | 0 | 10 | 30 |  | 1 | 1 | 3 |
| P27 |  | 60 | 80 | 90 |  | 10 | 27 | 50 |  | 10 | 20 | 40 |  | 1 | 2 | 3 |
| P28 |  | 70 | 90 | 100 |  | 10 | 23 | 30 |  | 0 | 10 | 30 |  | 1 | 1 | 2 |
| P29 |  | 70 | 90 | 100 |  | 10 | 20 | 30 |  | 0 | 10 | 30 |  | 1 | 1 | 2 |
| P30 |  | 70 | 90 | 100 |  | 10 | 30 | 50 |  | 0 | 10 | 30 |  | 1 | 2 | 5 |
| P31 |  | 60 | 80 | 90 |  | 10 | 33 | 60 |  | 10 | 20 | 40 |  | 1 | 2 | 6 |
| P32 |  | 70 | 90 | 100 |  | 10 | 23 | 40 |  | 0 | 10 | 30 |  | 1 | 1 | 2 |
| P33 |  | 70 | 90 | 100 |  | 10 | 28 | 50 |  | 0 | 10 | 30 |  | 1 | 1 | 3 |
